# The Effects of High Fiber Rye, Compared to Refined Wheat, on Gut Microbiota Composition, Plasma Short Chain Fatty Acids, and Implications for Weight Loss and Metabolic Risk Factors (the RyeWeight Study)

**DOI:** 10.3390/nu14081669

**Published:** 2022-04-17

**Authors:** Kia Nøhr Iversen, Johan Dicksved, Camille Zoki, Rikard Fristedt, Erik A. Pelve, Maud Langton, Rikard Landberg

**Affiliations:** 1 Department of Biology and Biological Engineering, Division of Food and Nutrition Science, Chalmers University of Technology, 412 96 Gothenburg, Sweden; kia.nohr@chalmers.se (K.N.I.); zoki@chalmers.se (C.Z.); rikfri@chalmers.se (R.F.); 2 Department of Animal Nutrition and Management, Swedish University of Agricultural Sciences, 750 07 Uppsala, Sweden; johan.dicksved@slu.se; 3 Department of Anatomy, Physiology and Biochemistry, Swedish University of Agricultural Sciences, 750 07 Uppsala, Sweden; erik.pelve@slu.se; 4 Department of Molecular Sciences, Swedish University of Agricultural Sciences, 750 07 Uppsala, Sweden; maud.langton@slu.se

**Keywords:** rye, wheat, cereal fiber, whole grain, weight loss, inflammation, metabolic risk markers, gut microbiota, short chain fatty acids

## Abstract

Consumption of whole grain and cereal fiber have been inversely associated with body weight and obesity measures in observational studies but data from large, long-term randomized interventions are scarce. Among the cereals, rye has the highest fiber content and high rye consumption has been linked to increased production of gut fermentation products, as well as reduced risks of obesity and metabolic disease. The effects on body weight and metabolic risk factors may partly be mediated through gut microbiota and/or their fermentation products. We used data from a randomized controlled weight loss trial where participants were randomized to a hypocaloric diet rich in either high fiber rye foods or refined wheat foods for 12 weeks to investigate the effects of the intervention on gut microbiota composition and plasma short chain fatty acids, as well as the potential association with weight loss and metabolic risk markers. Rye, compared to wheat, induced some changes in gut microbiota composition, including increased abundance of the butyrate producing *Agathobacter* and reduced abundance of *[Ruminococcus] torques* group, which may be related to reductions in low grade inflammation caused by the intervention. Plasma butyrate increased in the rye group. In conclusion, intervention with high fiber rye foods induced some changes in gut microbiota composition and plasma short chain fatty acid concentration, which were associated with improvements in metabolic risk markers as a result of the intervention.

## 1. Introduction

Consumption of whole grain and cereal fiber have been associated with improved body weight management and a lower risk of developing overweight and obesity, as well as a reduced risk of several co-morbidities, such as type-2 diabetes and cardiovascular diseases [1,2,3,4,5]. Lately, gut microbiota has emerged as a potential mediator of effects of diet on health and disease [6], both in terms of the ability of diet to affect the composition of the gut microbiota, but also the ability of gut microbiota to influence the response to dietary exposures [6,7,8].

Although dietary fiber is considered a key diet component to modulate gut microbiota [9,10], interventions with cereal products, the main sources of dietary fiber in diets worldwide [11], have shown limited ability to alter the composition of the gut microbiota [12,13,14]. This may be due to the fact that individuals respond differently to interventions and it may therefore be difficult to detect shifts in the microbial composition on a group level, but it could also be related to the resilience in the microbiota in response to high cereal fiber intake and because interventions with high cereal fiber have often been undertaken in populations with rather high habitual cereal fiber intake [15,16]. However, some studies have indicated that intervention with cereal fibers may increase the abundance of certain bacteria, such as *Bifidobacterium* and *Lactobacillus*, even in the context of an otherwise habitual diet [17,18,19].

An emerging area of interest is the ability of gut microbiota to influence clinical responses to specific dietary exposures. Differences in the compositions of gut microbiota may influence the utilization of specific compounds in the diet and in turn influence the response to the diet [20,21]. Studies have, for example, indicated that abundance of *Prevotella* and *Bacteroides* may be used to predict the response to dietary intervention with a high content of whole grain cereals, or specific dietary fiber from whole grain [22,23,24,25,26]. Such information may be useful to identify individuals or groups of individuals who may respond particularly well to a certain intervention and individuals who are unlikely to respond well and may benefit more from another type of intervention. Traditionally, dietary advice has been given on a population level. The recent findings on differential responses due to gut microbiota indicates that although certain dietary patterns can be considered healthy on a population level, there is a large variation in responses within the population, suggesting that targeting advice for specific groups and/or individuals may be a useful tool to improve the health of the entire population [27,28,29].

In this study, we investigated the effects of a dietary intervention on gut microbiota and plasma short chain fatty acids (SCFAs) and their potential roles as mediators of weight-loss induced by a hypocaloric diet rich in high fiber rye foods vs. refined wheat in a 12-week weight-loss trial. We also investigated if improvements in clinical risk markers caused by the intervention could be related to changes in gut microbiota and SCFAs in plasma and if baseline microbiota was associated with the response to the intervention.

## 2. Methods

The study design and procedures of the clinical study have been published in detail elsewhere and will therefore only be described briefly here [30]. The study was a randomized controlled parallel intervention examining the effect of high fiber rye cereal foods, compared with refined wheat foods, on body weight and body fat loss in the context of a hypocaloric diet. After a 2-week run-in period where all participants consumed refined wheat products, participants were randomized to consume either refined wheat products or high fiber rye products for 12 weeks (Supplementary Figure S1). During all 14 weeks, participants received dietary guidance from dieticians, aiming at a 500 kcal/day energy deficit to induce weight loss. Participants were instructed to avoid consumption of cereals not included in the study during all 14 weeks. At weeks 0, 6, and 12 of the parallel intervention phase, participants underwent a clinical examination, including anthropometric measurements, a dual energy x-ray absorptiometry (DXA) scan, and fasting blood sample collection. Before each clinical examination, participants collected a fecal sample and brought it to the clinic.

### 2.1. Ethical Considerations and Registration

The study was conducted in Uppsala, Sweden, between September 2016 and December 2018. The study was approved by the Ethical Review Board in Uppsala, conducted in accordance with the Declaration of Helsinki and registered at www.clinicaltrials.gov (Identifier: NCT03097237, accessed on 20 March 2022). All participants gave informed consent in writing before participation.

### 2.2. Participants, Randomization, and Blinding

Men and women aged 30–70 years, with body mass index (BMI) of 27–35 kg/m^2^ were eligible for participation. Exclusion criteria were history of chronic gastrointestinal disorders or major gastrointestinal surgery, use of antidiabetic drugs, thyroid disorder, untreated hypertension or hyperlipidemia, use of anti-obesity drugs/supplements or adherence to a weight loss regimen within the past 6 months. Furthermore, participants were required to lose ≥0.5 kg during the 2-week run-in period in order to be randomized into the 12-week parallel intervention phase. Participants who completed the 2-week run-in period were randomized 1:1 to receive either rye or wheat products for the 12-week parallel intervention phase. Participants were not blinded to their allocation due to the visual differences between the intervention products, but the nurses and technicians conducting the examinations were blinded. A detailed description of the screening procedures, inclusion and exclusion criteria, blinding, and randomization, has been published elsewhere [30].

### 2.3. Intervention Products

The intervention products consisted of breakfast cereals, soft bread, and crisp bread in both the rye and the wheat groups and were chosen to reflect the typical products available on the Swedish market. Breakfast cereals consisted of extruded puffs based on refined wheat or whole grain rye, rolled rye flakes, and wheat semolina. The soft rye bread was sliced whole grain rye bread, whereas the soft wheat bread consisted of portion-sized baguettes based on refined wheat. The rye group had a selection of 5 different whole grain rye crisp breads to choose from, while the wheat group was provided one type of crisp bread based on refined wheat. Participants were instructed to consume a fixed amount of products per day (650 kcal/day, corresponding to approximately 30–50% of energy intake) and record their intake in a pre-coded compliance journal. Additionally, alkylresorcinols were measured in plasma as a supporting measure of compliance [31]. The daily amount of rye products provided approximately 30 g dietary fiber/day, whereas the wheat products provided 8 g dietary fiber/day. Additional information regarding the intervention products can be found in Supplementary Tables S1 and S2.

### 2.4. Clinical Examination and Sample Collection

Participants arrived at the clinic after an overnight fast, bringing with them a fecal sample. Body weight was measured on a digital scale (Tanita BC-545N scale, Tanita Corporation, Tokyo, Japan) with the participant wearing light clothing. Participants underwent a total body DXA scan to determine body composition in terms of fat mass and lean body mass (Lunar Prodigy, GE Medical Systems, Chicago, IL, USA). Fasted blood samples were collected and were centrifuged, aliquoted, and stored in −80 °C until analysis after the completion of the study. Glucose, C-reactive protein (CRP), total cholesterol, low-density lipoprotein (LDL) cholesterol, high density lipoprotein (HDL) cholesterol, and triglycerides were measured in sodium heparin plasma, while insulin was measured in serum, at the Department of Clinical Chemistry at Uppsala University Hospital, Uppsala, Sweden [30]. Alkylresorcinols were measured in EDTA plasma as an objective marker of compliance [31,32].

Participants were provided with an EasySampler Faeces Kit (GP Medical Devices Ltd., Holstebro, Denmark), containing a fecal collection tube (Sarstedt AG & Co., Munich, Germany), and were asked to fill 2–3 spoonful of feces in the tube. Participants were instructed to store the sample in a cooling bag with frozen cooling blocks for a maximum duration of 24 h before delivering the sample to the clinic, or alternatively store the sample in their household freezer for up to three days before transporting it to the clinic in the cooling bag with frozen cooling blocks. At the clinic, the fecal samples were stored in −20 °C for a maximum of 7 days, before being transferred to −80 °C freezer for long-term storage.

### 2.5. SCFA in Plasma

A panel of 9 SCFAs were measured in heparin plasma at Chalmers University of Technology according to a method described by Han et al. [33], with the following described modifications: compounds of nine straight and branched-chain SCFAs, reagent-grade 3-nitrophenylhydrazine (3NPH)HCl (97%) and N-(3-dimethylaminopropyl)-N0-ethylcarbodiimide (EDC), high performance liquid chromatography (HPLC) grade pyridine, liquid chromatography-mass spectrometry (LC–MS) grade acetonitrile, LC–MS water and MeOH (Lichrosol) were purchased from Sigma–Aldrich (Saint Louis, MI, USA). All reagents and solvents were used for a maximum of 5 days to avoid contamination; 13C6-3NPH was custom synthesized by IsoSciences Inc. (King of Prussia, PA, USA) and used as internal standards for all SCFAs. A plasma volume of 10 μL was incubated with 60 μL 75% methanol, 10 μL 200 mM 3-NPH, and 10 μL 120 mM EDC-6% pyridine at ambient temperature for 45 min with gentle shaking. The reaction was quenched by addition of 10 μL of 200 mM quinic acid (15 min with gentle shaking at ambient temperature). The samples were centrifuged at 15,000× *g* for 5 min and the supernatant moved to a new tube. The samples were made up to 1 mL by 10% methanol in water and again centrifuged at 15,000× *g* for 5 min. A volume of 100 μL of the derivatized (12C) sample was mixed with 100 μL of labelled (13C) internal standard. The samples were analyzed by a 6500+ QTRAP triple-quadrupole mass spectrometer (AB Sciex, Stockholm, Sweden), which was equipped with an APCI source and operated in the negative-ion mode. Chromatographic separations were performed on a Phenomenex Kinetix Core-Shell C18 (2.1, 100 mm, 1.7 um 100 Å) UPLC column with SecurityGuard ULTRA Cartridges (C18 2.1 mm ID). LC–MS grade water (100% solvent A) and acetonitrile (100% solvent B) was the mobile phases for gradient elution. The column flow rate was 0.4 mL/min and the column temperature was 40 °C, the autosampler was kept at 5 °C. LC starting conditions at 0.5% B, held for 3 min, 3 min 2.5% B ramping linearly to 17% B at 6 min, then to 45% B at 10 min and 55% B at 13 min. Followed by a flush (100% B) and recondition (0.5% B), total runtime 15 min. The MRM transitions were optimized for the analytes one-by-one by direct infusion of the derivatives containing 50 mM of each fatty acid. The Q1/Q3 pairs were used in the MRM scan mode to optimize the collision energies for each analyte, and the two most sensitive pairs per analyte were used for the subsequent analyses. The retention time window for the scheduled MRM was 1 min for each analyte. The two MRM transitions per analyte, the Q1/Q3 pair that showed the higher sensitivity was selected as the MRM transition for quantitation. The other transition acted as a qualifier for the purpose of verification of the identity of the compound.

### 2.6. Microbiota Analysis

Total DNA was extracted from the fecal samples using QIAamp Fast DNA Stool mini kit (Qiagen, Hilden, Germany) according to the protocol from the manufacturer, with the exception that the cell walls were mechanically disrupted with 0.1 mm Zirconium/Silica beads (Biospec products, Bartlesville, OK, USA) 2 × 60 s using a Precellys Evolution, (Bertin Technologies, Montigny-le-Bretonneux, France).

Amplicons from the V3 and V4 regions of the 16S ribosomal RNA gene were generated from the extracted DNA using the primers 341F and 805R. For the polymerase chain reactions (PCRs), Phusion^®^ High-Fidelity PCR Master Mix (New England Biolabs, Ipswich, MA, USA) were used and the PCR products were purified with Qiagen Gel Extraction Kit (Qiagen, Hilden, Germany) and quantified with Qubit^®^3.0 Fluorometer. The final libraries were generated with NEBNext^®^ UltraTM DNA Library Prep Kit that incorporated barcodes and adaptors. The amplicons were then sequenced on the Illumina platform at Novogene (Beijing, China).

The raw demultiplexed reads from the sequencing were processed using the DADA2 pipeline to denoise (with the following parameters used in the filterAndTrim-step: maxN = 0, maxEE = c(2,2), truncQ = 2, rm.phix = TRUE, compress = TRUE), dereplicate reads, merge pair end reads and remove chimeras [34]. The tables of amplicon sequence variants (ASVs) were assigned to reference sequences using the naive Bayesian classifier called with the assignTaxonomy command [35] against the SILVA rRNA database [36], release 138, formatted for DADA2 by B. Callahan (https://benjjneb.github.io/dada2/training.html, accessed on 1 February 2021). The Phyloseq R package was used to construct ASV frequency tables used for subsequent statistical analysis [37]. Alpha diversity, in terms of Shannon’s diversity index, Chao1 index, and Simpson’s diversity index, was calculated based on the ASV data.

### 2.7. Statistical Analysis

Sample size was estimated based on the primary outcomes of the study, body weight, and body fat, with a power of 80% and alpha of 2.5%. For secondary outcomes, alpha was set to 5% [30]. Analysis of gut microbiota and plasma SCFAs were considered explorative investigations and were not subjected to any power calculation.

Before the statistical analysis, data on gut microbiota composition was filtered so that only bacteria (characterized on genus level) present in at least 50% of the samples were included in the analysis. This resulted in 110 bacteria being included in the analysis. Effect of the dietary intervention on the relative abundance of the bacteria on the genus level and SCFA concentration were evaluated using the PROC MIXED procedure in SAS statistical software version 9.4 (SAS institute, Cary, NC, USA). Week and week x diet were included as fixed factors, and a REPEATED statement specifying an unstructured correlation structure with the week as the repeated factor was included. Models compared the groups at week 6/week 12 and were adjusted for baseline (week 0). Data were log transformed before analysis and zero counts were replaced by 10^−^^5^ before transformation. False discovery rate (FDR) correction was applied to the comparisons of the bacterial abundance between the groups, but both raw-***p***-values and FDR-corrected *p*-values are reported due to the explorative nature of the analysis. Correlations were calculated using the PROC CORR procedure, specifying Spearman’s rank correlation. Figures were made using the ggplot2 package in Rstudio version 4.0.4 (R Foundation for Statistical Computing, Vienna, Austria).

## 3. Results

In total, 242 participants were randomized into the 12-week parallel intervention period and 207 of these completed the 12-week intervention (Supplementary Figure S2). All 207 participants provided a complete set of fecal samples (weeks 0, 6, and 12) and are all included in this analysis. Baseline characteristics can be found in Table 1.

Over the 12 weeks, participants in both groups lost body weight and body fat, but the rye group lost more than the wheat group [30]. Furthermore, the rye group reduced CRP at week 6 (mean concentration, rye vs. wheat: 1.2 mg/L vs. 1.5 mg/L, *p* = 0.02) and week 12 (mean concentration, rye vs. wheat: 1.1 mg/L vs. 1.6 mg/L, *p* < 0.01), seemingly independent of weight loss as the analysis was adjusted for the change in body weight and no correlation between the change in body weight and CRP was found (r < 0.1, *p* > 0.1) [30]. Additionally, the wheat group increased LDL cholesterol after 6 weeks of intervention, though the effect on LDL cholesterol had diminished at week 12. There were no effects of the intervention on total cholesterol, HDL cholesterol, triglycerides, glucose, or insulin [30]. The compliance was high in both the rye and the wheat group with 94–95% of the prescribed amount of products being consumed and a significant increase in plasma alkylresorcinol concentration among participants in the rye group [30]. As intended, both intervention groups reduced their energy intake over the course of the intervention (approximately 150–200 kcal/day, compared to baseline), but the energy intake did not differ between the groups at any time point (*p* > 0.4) [30].

### 3.1. Effect of the Intervention of Microbiota Composition

**Figure 1:**
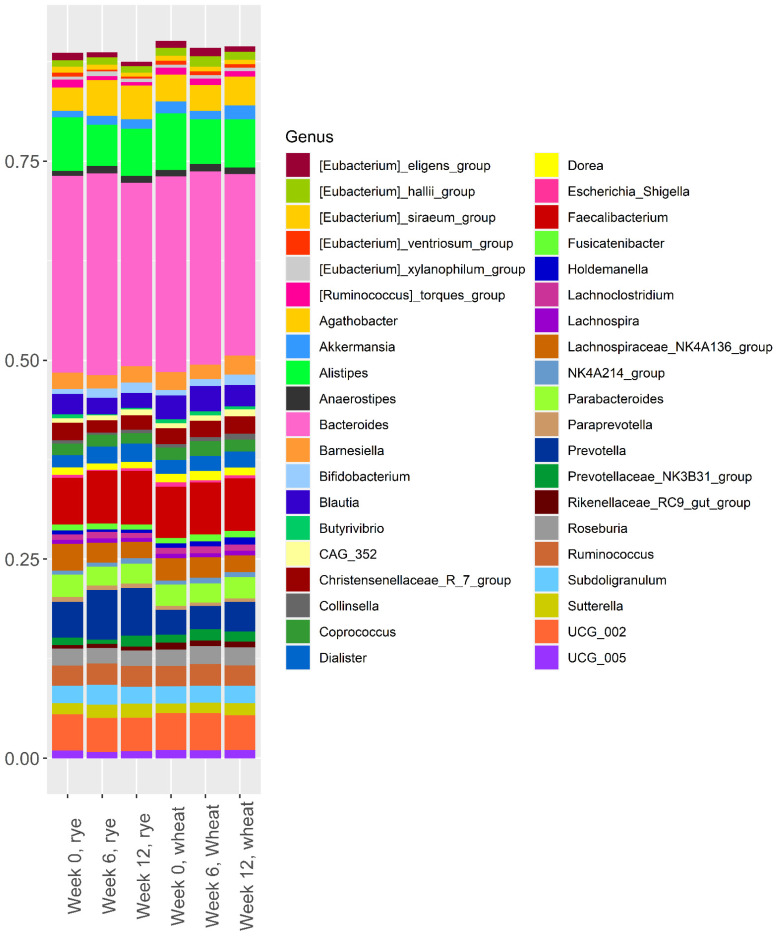
Relative abundance of the 40 most abundant bacteria at week 0, week 6, and week 12 in the rye group and the wheat group.

The relative abundance of the 40 most abundant bacteria in the two intervention groups at weeks 0, 6, and 12 are shown in Figure 1. For clarity, only the 40 most abundant bacteria are included in Figure 1, but all 110 bacteria that remained after filtering are included in the analysis presented in Table 2 and Supplementary Table S3.

Figure 1 clearly shows that *Bacteroides* was the most abundant bacteria in both groups, at all time-points. Furthermore, *Alistipes, Faecalibacterium, Prevotella*, and *UCG_002* also had a high abundance at all time-points. Visual inspection of Figure 1 does not reveal any clear shift in gut microbiota composition over the course of the intervention. There was no difference between the rye group and the wheat group in terms of alpha diversity at any point during the 12-week intervention (Supplementary Figure S3).

Analysis of the effect of the intervention on the relative abundance of the 110 bacteria remaining after filtering revealed a difference between the groups at either week 6 or week 12 in 45 of the bacteria; however, only 8 of these remained significant after FDR correction (Table 2). The rye diet caused a decreased abundance of (*Ruminococcus*) *torques *group, (*Eubacterium*) *ventriosum* group, *Anaerofilum*, and *Holdemania* while the abundance of *Agathobacter*, *UCG-003*, and *Haemophilus* increased, compared to the wheat group, over the course of the intervention. *Anaerotruncus* decreased in both groups, but more so in the rye group (Table 2). *Prevotella* abundance increased numerically in the rye group, but there was no significant difference between the groups at week 6 or week 12 due to a baseline difference in abundance (Figure 1). Similarly, *Bifidobacterium* abundance increased in both groups, but were not different between the groups after FDR correction (Supplementary Table S3).

Correlations between the changes in relative abundance of the eight bacteria that differed between the groups after FDR correction and changes in clinical outcomes are presented in Table 3. Changes in body fat mass and body fat percentage in the rye group correlated negatively with change in the abundance of *Holdemania*. Change in body fat mass in the wheat group was positively correlated with change in abundance of *UCG-003*; however, the rye diet induced a larger increase in *UCG-003* abundance than the wheat group. Despite no effect of the wheat diet on the inflammation marker CRP, changes in CRP correlated negatively with change in the abundance of the (*Ruminococcus*) *torques* group and (*Eubacterium*) *ventriosum* group, indicating that an increase in abundance was associated with a decrease in CRP in the wheat group.

### 3.2. Baseline Microbiota and Change in Body Weight and Body Fat over 12 Weeks

**Figure 2:**
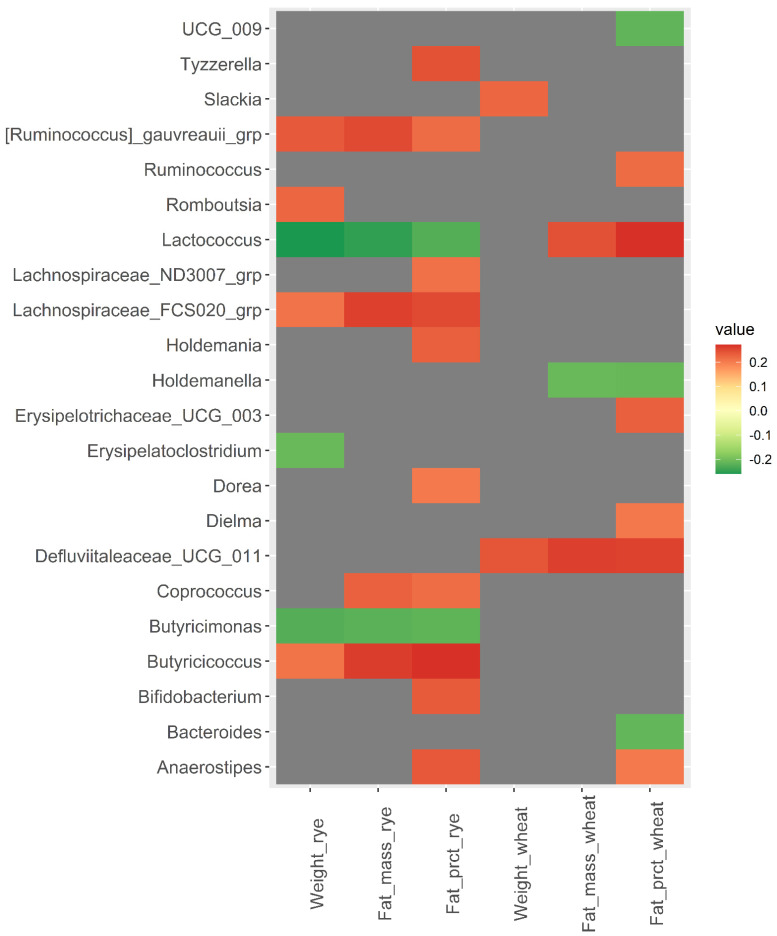
Heatmap of correlations (Spearman’s *rho*) between bacterial abundance at baseline and changes in body weight, body fat mass, and body fat percentage over 12 weeks within the wheat group and the rye group. Only significant correlations with Spearman’s *rho* ≥ |0.200| are included in the heatmap, while correlations for all 110 bacteria are shown in Supplementary File S2.

Correlation between the relative abundance of the 110 bacteria included in the analysis at baseline and change in body weight and body fat from baseline to week 12 revealed some interesting correlations (Figure 2 and Supplementary File S2). Baseline abundance of *Lactococcus* was negatively correlated with fat mass and fat percentage in the rye group (*rho* = −0.25 and *p* = 0.009, *rho* = −0.23, *p* = 0.019), but positively correlated in the wheat group (*rho* = 0.25 and *p* = 0.015, *rho* = 0.27 and *p* = 0.007). *[Ruminococcus] gnauvreauii* group and *Butyricicoccus* were positively correlated with change in body weight, body fat mass, and body fat percentage in the rye group (*rho* ≥ 0.21, *p* < 0.015), while *Butyricimonas* was negatively correlated with change in body weight, body fat, and body fat percentagewithin the rye group (*rho* ≤ −0.22, *p* < 0.020). *Defluviitaleaceae UCG-011* was positively correlated with change in body weight and body fat in the wheat group (*rho* ≥ 0.24, *p* < 0.018).

### 3.3. Baseline Microbiota and Changes in Metabolic Risk Markers over 12 Weeks

**Figure 3:**
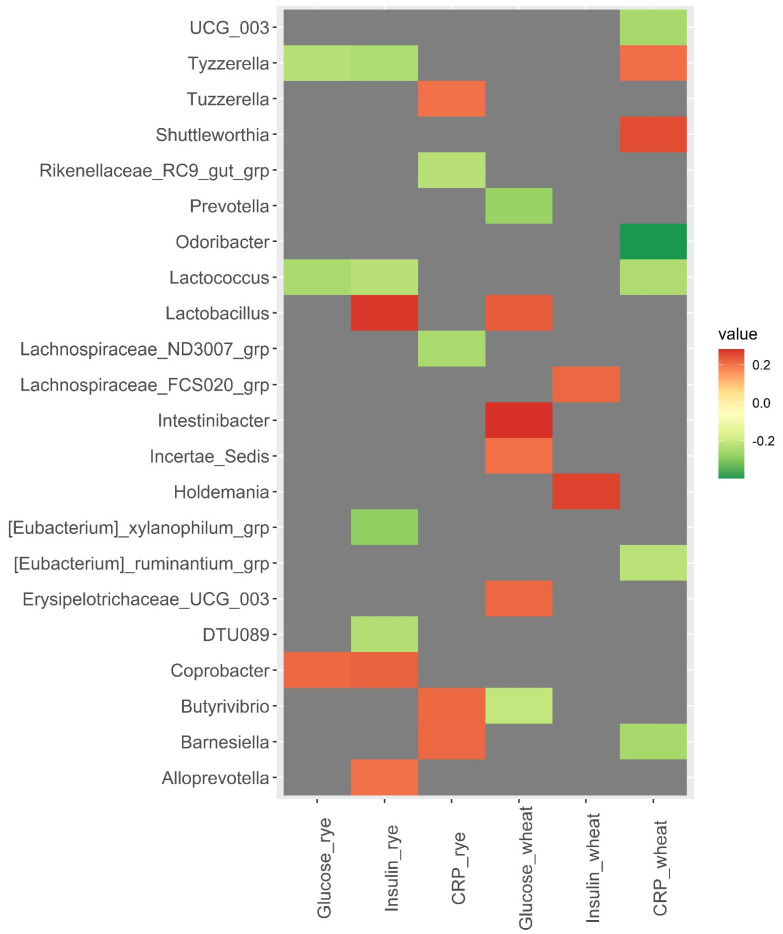
Heatmaps of correlations (Spearman’s *rho*) between bacterial abundance at baseline and changes in glucose, insulin, and C-reactive protein over 12 weeks within the wheat group and the rye group. Only significant correlations with Spearman’s *rho* ≥ |0.200| are included in the heatmap, while correlations for all 110 bacteria are shown in Supplementary File S2. Abbreviations: CRP, C-reactive protein.

**Figure 4:**
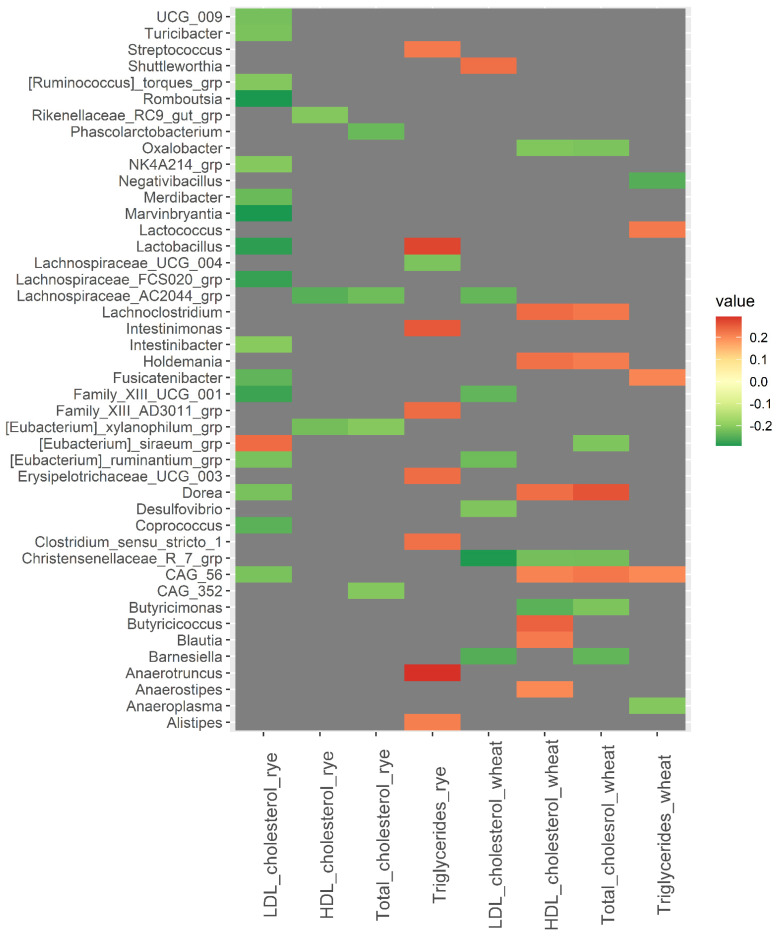
Heatmaps of correlations (Spearman’s *rho*) between bacterial abundance at baseline and changes in blood lipids over 12 weeks within the wheat group and the rye group. Only significant correlations with Spearman’s *rho* ≥ |0.200| are included in the heatmap, while correlations for all 110 bacteria are shown in Supplementary File S2. Abbreviations: LDL, low-density lipoprotein; HDL, high density lipoprotein.

A strong correlation was observed between baseline abundance of *Odoribacter* and changes in CRP in the wheat group (*rho* = −0.40, *p* = <0.001) (Figure 3). Additionally, *Barnesiella* was positively correlated with changes in CRP in the rye group (*rho* = 0.22, *p* = 0.024) and negatively correlated with changes in CRP in the wheat group (*rho* = −0.25, *p* = 0.011). *Tyzzerella* and *Lactococcus* were both inversely correlated with changes in insulin and glucose in the rye group (*rho* ≤ −0.23, *p* < 0.020). *Christensenellaceae R7* group was inversely correlated with changes in total cholesterol, HDL cholesterol, and LDL cholesterol in the wheat group (*rho* ≤ −0.22, *p* < 0.028) (Figure 4). However, results related to metabolic risk markers should be interpreted with caution, since these results are likely confounded to some degree by the weight loss caused by the intervention.

### 3.4. SCFAs in Plasma

Among the SCFAs, butyrate increased in the rye group and plasma concentration was higher in the rye group than in the wheat group after 6 and 12 weeks of intervention (Table 4). The absolute differences were small though. Acetic acid concentration decreased in the rye group and was lower than the wheat group after 6 weeks of intervention, but the difference had attenuated at week 12 as the concentration had also decreased in the wheat group (Table 4).

Changes in butyrate and propionate were inversely correlated with changes in body fat percentage in the rye group (*rho* ≥ −0.19, *p* < 0.049), but not in the wheat group (Supplementary Table S4). Succinic acid was inversely correlated with changes in body weight, body fat mass, and body fat percentage in the wheat group (*rho* < −0.21, *p* < 0.0345), as well as inversely correlated with changes in CRP in the rye group (*rho* = −0.21, *p* = 0.0284) (Supplementary Table S4).

## 4. Discussion

In this study, we found that a 12-week intervention with high fiber rye foods, compared to refined wheat foods, as part of a hypocaloric diet, induced some alterations in gut microbiota composition, although without any apparent systematic shifts in the microbial community composition. Some of the changes in gut microbiota composition appeared to be associated with reductions in CRP induced by the rye diet. Moreover, the rye diet was associated with modestly, but significantly higher concentration of butyrate in plasma. Furthermore, exploratory analysis showed that the presence of some bacteria at baseline correlated with changes in body weight and body fat reduction induced by the intervention.

We found that the abundance of the butyrate producing bacteria *Agathobacter* increased in the rye group, while the abundance remained unchanged in the wheat group. *Agathobacter* has previously been associated with intake of oat-based beta-glucan rich bread [38], and while the beta-glucan in rye is lower than oat, rye contains a large amount of other fermentable fibers, which is likely the cause of increased abundance of fiber degrading bacteria. *Agathobacter* is known to produce butyrate and may have contributed to the increase in plasma butyrate concentration in the rye group [39,40]. Butyrate has been associated with reduced appetite, possibly through increase in GLP-1, and subsequently lower energy intake and reduced body weight [41,42,43,44], which could have contributed to the larger weight loss in the rye group, compared to the wheat group. Fecal SCFAs has been shown to be higher in overweight and obese subjects, compared to lean subjects [45,46], which may seem counterintuitive if SCFAs are involved in regulating energy balance and associated with increased satiety and energy expenditure. However, a recent study comparing fecal and plasma SCFAs found that plasma and fecal SCFAs are not particularly well correlated and that plasma SCFAs were inversely correlated with BMI and positively correlated with GLP-1 [47]. This indicates that plasma SCFAs are stronger determinants for the proposed health effects of SCFAs than fecal SCFAs.

Furthermore, we found that the rye diet resulted in a lower abundance of *(Ruminococcus) torques* group, compared to the wheat group. Bacteria of this genera are known to have a negative impact on gut barrier integrity, likely due to degradation of the mucus barrier [48,49,50]. Decreased gut barrier integrity has been associated with low grade inflammation and it is possible that the reduced plasma CRP concentration following the rye-based intervention might partially be mediated through improved gut barrier and that reduction of the *(Ruminococcus) torques* group abundance may have contributed to such an effect [51,52,53]. Butyrate, which was increased in the rye group, has also been suggested to reduce low-grade inflammation, which may also be mediated by improved gut barrier integrity [54]. It should be mentioned that the participants in the study were relatively healthy and there was no consensus on the cut-offs for CRP in relation to cardiovascular disease risk, so the findings should be interpreted with care. However, CRP concentration of 1–3 mg/l has been suggested to be interpreted as an intermediate risk and even small reduction in CRP in a seemingly healthy population could be valued in relation to prevention on disease [55,56].

Interestingly, *Holdemania* was reduced in the rye group over the course of the 12-week intervention period, while the change in abundance was inversely correlated with change in body fat in the same group of participants, which seems conflicting. Arumugam et al. found *Holdemania* to co-occur within the Prevotella-driven enterotype, while another study has found *Holdemania* to be associated with poorer metabolic health and an in vitro study found *Holdemania* to be unaffected by different fiber substrates [57,58,59]. One study in patients with irritable bowel syndrome found that a rye bread based on a sourdough specifically developed to result in a bread with low content of FODMAPs (fermentable oligo-, di-, mono-saccharides and polyols) reduced abundance of *Holdemania* after 4 weeks of intervention [60]. However, traditional rye bread did not result in a significant decrease in *Holdemania* and irritable bowel disease has been associated with altered gut microbiota, so the results should be interpreted with care [60,61]. It seems that little is known about the function of *Holdemania* in the human gut and its potential link to human health. It is possible that different species and strains of *Holdemania* have different functions in the gut and different effects on the host, which could explain why the few existing results point in different directions.

We found that baseline abundance of *Lactococcus* was inversely correlated with changes in body weight and body fat in the rye group, while being positively correlated in the wheat group. Interestingly, we did not find *Prevotella* to be correlated with changes in body weight or body fat reduction, despite several other studies conducted in similar populations finding a high *Prevotella* abundance being correlated with weight loss on similar fiber rich diets [22,25]. Previous studies have also found relatively high numbers of study participants to have a *Prevotella* abundance below the detection limit, but we found that almost all participants (97%) had detectable *Prevotella* at baseline [22,24].

An important limitation of the present study is that it included a 2-week run-in period where all participants consumed the wheat-based products, before participants underwent the baseline examination and initiated the 12-week parallel intervention period and were randomized to either rye or wheat products. Therefore, the baseline microbiota in this study may not reflect the usual gut microbiota of the participants before the intervention since we do not know if the run-in period induced any changes in the microbiota. We observed some changes in microbiota composition in the wheat group over the 12-week parallel intervention period, which could indicate that the wheat diet can affect the gut microbiota and may have induced changes over the run-in period. The bacteria for which we found associations between baseline abundance and response in terms of weight loss did not differ between the groups during the 12-week period and were seemingly unaffected by the intervention. Furthermore, as the study was designed as a weight loss study, it cannot be ruled out that any changes in metabolic risk markers may be confounded by weight loss and these results should therefore be interpreted with caution. Additionally, as the intervention products were chosen to reflect the products typically available on the Swedish market, the products did not only differ in their cereal source, but also in the dietary fiber content. Therefore, it is difficult to disentangle the effects of cereal source and dietary fiber per se and the results should be interpreted with this in mind. The present study relied on data from 16s rRNA gene sequencing methodology with taxonomy resolution limited to the genus level. A deeper level of resolution may give more insight into the link between microbiota and response to the intervention. Lastly, it is important to remember that the current study was an exploratory investigation of data from a study that was primarily designed to investigate the effect of the dietary intervention on weight-loss. Although our study was large compared to other studies investigating the effects of cereals and dietary fiber on gut microbiota and, therefore, it is likely that even small differences in gut microbiota could be detected [17,18], there is a need for studies designed specifically to investigate the role of the gut microbiota in order to establish causality.

A strength of the study is that it was characterized by a high rate of compliance, estimated by both objective and subjective markers of compliance, and used comprehensively characterized intervention products. We found that the intervention affected more bacterial groups than other similar investigations have found, which could be a result of a larger sample size and thereby increased statistical power [12,13].

## 5. Conclusions

In conclusion, a dietary intervention with high fiber rye products as part of a hypocaloric diet induced some changes in gut microbiota composition and plasma SCFA concentration, which appeared to be associated with weight loss and improvement in metabolic risk markers induced by the intervention. Furthermore, baseline abundance of some bacteria correlated oppositely with changes in body weight within the two intervention groups, though this should be interpreted with care as the study design was not optimal for evaluating the association between baseline gut microbiota and response to the intervention. The results of this study support the growing body of evidence suggesting the gut microbiota may be a mediator of the health promoting effect of whole grains and cereal fiber; however, studies specifically designed to investigate this are needed in order to establish causality.

## Figures and Tables

**Table 1:** Baseline characteristics.

	Rye Group (*n* = 108)	Wheat Group (*n* = 99)
Sex (*n*, females/males)	61/47	62/37
Age (years)	56.8 ± 9.4	57.3 ± 9.6
Body weight (kg)	88.8 ± 12.8	89.1 ± 12.3
BMI (kg/m^2^)	29.8 ± 2.5	30.3 ± 2.5
Body fat mass (kg)	34.0 ± 6.5	35.8 ± 7.2
Body fat percentage	38.9 ± 6.8	40.7 ± 7.0
CRP (mg/L)	1.5 (0.7; 3.0)	1.5 (0.9; 2.4)
Total cholesterol (mmol/L)	4.7 ± 0.9	4.8 ± 1.0
LDL cholesterol (mmol/L)	3.1 ± 0.8	3.1 ± 0.8
HDL cholesterol (mmol/L)	1.4 ± 0.3	1.4 ± 0.3
Triglycerides (mmol/L)	1.0 (0.9; 1.3)	1.1 (0.9; 1.3)
Glucose (mmol/L)	5.5 ± 0.5	5.6 ± 0.6
Insulin (mIU/L)	8.7 (6.6; 12.3)	10.4 (7.3; 13.2)

Data are mean ± sd or median (IQR). Abbreviations: BMI, body mass index; CRP, C-reactive protein; LDL, low density lipoprotein; HDL high density lipoprotein.

**Table 2:** Relative abundance of bacteria that differed significantly between the rye and the wheat group at either week 6 and/or week 12 after correction for multiple testing. In total, 110 bacteria were tested, 45 differed significantly between the groups before FDR correction, and 8 remained significant after FDR correction. The 8 bacteria that differed significantly after FDR correction are included in this table while the remainder of the 45 can be found in Supplementary Table S3.

					Raw *p*-Value/FDR Corrected *p*-Value **	
		Week 0 *	Week 6 *	Week 12 *	Week 6	Week 12
*Agathobacter*	Wheat	2.70 (2.36;3.10)	2.79 (2.49;3.14)	3.07 (2.72;3.46)	<0.001/0.010	0.033/0.985
	Rye	2.32 (2.04;2.65)	3.66 (3.28;4.10)	3.46 (3.08;3.88)		
*[Ruminococcus]* *torques* group	Wheat	0.69 (0.61;0.79)	0.62 (0.55;0.71)	0.60 (0.54;0.68)	<0.001/0.010	<0.001/0.010
	Rye	0.73 (0.65;0.83)	0.42 (0.38;0.48)	0.38 (0.34;0.43)		
[*Eubacterium*] *ventriosum* group	Wheat	0.37 (0.32;0.43)	0.30 (0.26;0.35)	0.27 (0.24;0.31)	<0.001/0.010	<0.001/0.010
	Rye	0.34 (0.30;0.40)	0.20 (0.17;0.23)	0.18 (0.16;0.20)		
*Anaerotruncus*	Wheat	0.009 (0.007;0.012)	0.008 (0.006;0.011)	0.006 (0.005;0.008)	<0.001/0.010	0.004/0.367
	Rye	0.006 (0.005;0.008)	0.003 (0.003;0.004)	0.003 (0.003;0.004)		
*Anaerofilum*	Wheat	0.008 (0.006;0.011)	0.006 (0.005;0.008)	0.007 (0.005;0.008)	0.006/0.586	<0.001/0.032
	Rye	0.008 (0.006;0.010)	0.004 (0.003;0.005)	0.004 (0.003;0.005)		
*UCG-003*	Wheat	0.27 (0.24;0.32)	0.29 (0.25;0.33)	0.37 (0.32;0.42)	<0.001/0.010	0.010/0.874
	Rye	0.27 (0.23;0.31)	0.40 (0.35;0.46)	0.45 (0.40;0.51)		
*Holdemania*	Wheat	0.006 (0.005;0.008)	0.004 (0.003;0.005)	0.006 (0.005;0.008)	0.0129/0.996	<0.001/0.010
	Rye	0.006 (0.005;0.008)	0.002 (0.002;0.003)	0.003 (0.002;0.004)		
*Haemophilus*	Wheat	0.03 (0.02;0.04)	0.05 (0.04;0.06)	0.05 (0.04;0.07)	<0.001/0.011	<0.001/0.032
	Rye	0.04 (0.03;0.06)	0.11 (0.08;0.14)	0.11 (0.08;0.14)		

* Relative abundance in % as geometric mean and 95% CI (raw unadjusted data, back transformed from log scale). ** Linear mixed model, adjusted for baseline abundance, data were log transformed before analysis. *n*(rye/wheat) = 108/99.

**Table 3:** Correlations between the change in relative abundance of the eight bacteria included in Table 2 and the changes in clinical outcomes over the 12-week intervention period.

	ΔWeight	ΔFat Mass	ΔFat%	ΔCRP	ΔLDL Cholesterol	ΔHDL Cholesterol	ΔTriglyceride	ΔTotal Cholesterol	ΔGlucose	ΔInsulin
Δ*Agathobacter*										
- Rye group	0.104 (0.284)	0.082 (0.401)	0.058 (0.552)	0.115 (0.236)	0.048 (0.622)	−0.051 (0.788)	−0.136 (0.160)	−0.080 (0.410)	0.205 (0.034)	0.1630 (0.092)
- Wheat group	−0.069 (0.498)	0.010 (0.921)	0.028 (0.784)	0.0352 (0.730)	−0.038 (0.709)	−0.032 (0.757)	0.017 (0.867)	−0.022 (0.828)	0.170 (0.093)	0.159 (0.116)
- Pooled groups	−0.033 (0.640)	−0.005 (0.942)	−0.005 (0.939)	0.021 (0.760)	−0.025 (0.717)	−0.063 (0.371)	−0.055 (0.429)	−0.066 (0.348)	0.178 (0.011)	0.155 (0.025)
Δ*[Ruminococcus] torques* group										
- Rye group	0.045 (0.642)	−0.052 (0.595)	−0.116 (0.233)	0.102 (0.292)	0.135 (0.163)	0.105 (0.742)	0.057 (0.561)	0.027 (0.785)	−0.018 (0.851)	−0.015 (0.882)
- Wheat group	0.013 (0.901)	0.065 (0.523)	0.032 (0.751)	−0.242 (0.016)	−0.031 (0.760)	−0.096 (0.346)	−0.243 (0.016)	−0.177 (0.080)	−0.243 (0.015)	0.064 (0.530)
- Pooled groups	0.056 (0.420)	0.034 (0.626)	−0.013 (0.856)	−0.002 (0.974)	0.081 (0.248)	−0.026 (0.714)	−0.124 (0.074)	−0.065 (0.350)	−0.126 (0.070)	0.016 (0.824)
Δ*[Eubacterium] ventriosum* group										
- Rye group	−0.042 (0.665)	−0.097 (0.319)	−0.120 (0.216)	−0.119 (0.219)	−0.055 (0.570)	0.122 (0.732)	−0.066 (0.497)	0.019 (0.848)	0.074 (0.445)	0.133 (0.171)
- Wheat group	−0.069 (0.498)	−0.045 (0.659)	−0.079 (0.438)	−0.286 (0.004)	0.046 (0.650)	0.032 (0.754)	−0.117 (0.249)	0.027 (0.788)	0.036 (0.726)	0.084 (0.408)
- Pooled groups	−0.021 (0.769)	−0.043 (0.534)	−0.070 (0.316)	−0.121 (0.081)	0.022 (0.750)	0.059 (0.399)	−0.104 (0.137)	0.029 (0.680)	0.064 (0.360)	0.119 (0.088)
Δ *Anaerotruncus*										
- Rye group	−0.120 (0.217)	−0.107 (0.272)	−0.093 (0.337)	0.008 (0.931)	−0.154 (0.112)	−0.030 (0.405)	−0.254 (0.008)	−0.108 (0.267)	−0.035 (0.719)	−0.145 (0.133)
- Wheat group	−0.142 (0.161)	−0.160 (0.113)	−0.155 (0.124)	−0.097 (0.339)	−0.009 (0.933)	0.170 (0.093)	−0.131 (0.196)	0.099 (0.328)	−0.116 (0.251)	0.123 (0.224)
- Pooled groups	−0.113 (0.104)	−0.113 (0.104)	−0.105 (0.132)	−0.021 (0.769)	−0.069 (0.321)	0.052 (0.453)	−0.192 (0.006)	−0.005 (0.949)	−0.065 (0.349)	−0.016 (0.819)
Δ *Anaerofilum*										
- Rye group	−0.002 (0.987)	−0.045 (0.643)	−0.056 (0.567)	0.050 (0.607)	0.023 (0.817)	0.037 (0.917)	−0.105 (0.279)	0.018 (0.850)	−0.039 (0.689)	0.036 (0.708)
- Wheat group	0.005 (0.961)	−0.022 (0.827)	0.003 (0.973)	0.093 (0.360)	0.004 (0.966)	0.114 (0.263)	0.058 (0.571)	0.093 (0.360)	−0.072 (0.477)	0.014 (0.894)
- Pooled groups	0.036 (0.611)	−0.005 (0.944)	0.003 (0.961)	0.113 (0.105)	0.050 (0.477)	0.090 (0.195)	−0.037 (0.600)	0.068 (0.333)	−0.045 (0.523)	0.028 (0.689)
Δ *UCG−003*										
- Rye group	−0.139 (0.153)	−0.124 (0.201)	−0.088 (0.367)	0.052 (0.595)	0.123 (0.204)	0.037 (0.706)	0.089 (0.357)	0.109 (0.263)	−0.112 (0.248)	−0.136 (0.161)
- Wheat group	0.154 (0.129)	0.208 (0.039)	0.180 (0.075)	−0.045 (0.656)	−0.006 (0.954)	−0.178 (0.078)	0.145 (0.153)	−0.115 (0.259)	−0.041 (0.689)	−0.009 (0.931)
- Pooled groups	−0.030 (0.666)	−0.001 (0.985)	0.005 (0.943)	−0.040 (0.566)	0.053 (0.451)	−0.079 (0.257)	0.123 (0.077)	0.003 (0.964)	−0.091 (0.194)	−0.088 (0.205)
Δ *Holdemania*										
- Rye group	−0.157 (0.106)	−0.215 (0.025)	−0.267 (0.005)	0.012 (0.899)	0.035 (0.717)	−0.054 (0.658)	−0.011 (0.914)	0.049 (0.615)	0.063 (0.519)	0.081 (0.405)
- Wheat group	0.173 (0.087)	0.173 (0.086)	0.138 (0.173)	0.131 (0.196)	0.096 (0.344)	−0.003 (0.976)	−0.134 (0.186)	0.008 (0.940)	−0.093 (0.360)	−0.063 (0.538)
- Pooled groups	0.049 (0.480)	0.012 (0.865)	−0.037 (0.599)	0.128 (0.065)	0.088 (0.206)	0.044 (0.526)	−0.095 (0.172)	0.038 (0.590)	−0.007 (0.915)	0.0206 (0.768)
Δ *Haemophilus*										
- Rye group	−0.180 (0.062)	−0.124 (0.203)	−0.083 (0.393)	0.068 (0.484)	0.007 (0.939)	−0.019 (0.343)	−0.009 (0.930)	−0.018 (0.856)	−0.059 (0.543)	−0.064 (0.514)
- Wheat group	0.101 (0.322)	0.167 (0.099)	0.179 (0.077)	−0.055 (0.591)	−0.031 (0.762)	−0.107 (0.294)	0.019 (0.851)	−0.079 (0.436)	−0.175 (0.083)	−0.061 (0.552)
- Pooled groups	−0.082 (0.240)	−0.028 (0.685)	0.003 (0.962)	−0.028 (0.687)	−0.023 (0.743)	−0.121 (0.082)	0.019 (0.791)	−0.052 (0.460)	−0.105 (0.132)	−0.073 (0.295)

Data are Spearman’s *rho* (*p*-value). Significant correlations are marked in bold font. Abbreviations: CRP, C-reactive protein; LDL, low density lipoprotein; HDL, high density lipoprotein.

**Table 4:** Plasma short chain fatty acid concentration (µM).

		Week 0 *	Week 6 *	Week 12 *	*p*-Value Week 6 **	*p*-Value Week 12 **
Formic acid	Wheat	87.5 (81.6; 93.8)	84.5 (79.0; 90.3)	88.2 (81.6; 95.2)	0.5218	0.7776
	Rye	84.3 (78.8; 90.1)	84.94 (79.7; 90.6)	87.6 (81.4; 94.3)		
Acetic acid	Wheat	114.5 (100.6; 130.4)	95.3 (82.7; 109.8)	95.2 (83.0; 109.3)	0.0259	0.1989
	Rye	111.8 (98.7; 126.6)	114.9 (100.4; 131.5)	105.4 (92.4; 120.3)		
Propionic acid	Wheat	0.65 (0.56; 0.74)	0.62 (0.54; 0.71)	0.62 (0.53; 0.71)	0.2014	0.1640
	Rye	0.60 (0.53; 0.69)	0.67 (0.59; 0.77)	0.68 (0.59; 0.78)		
Butyric acid	Wheat	0.84 (0.68; 1.02)	0.69 (0.57; 0.83)	0.78 (0.63; 0.95)	<0.0001	0.0270
	Rye	0.68 (0.56; 0.82)	0.99 (0.83; 1.19)	0.93 (0.77; 1.12)		
Isobutyric acid	Wheat	0.17 (0.15; 0.19)	0.16 (0.14; 0.18)	0.17 (0.15; 0.18)	0.8106	0.3958
	Rye	0.16 (0.14; 0.17)	0.16 (0.14; 0.17)	0.15 (0.14; 0.17)		
Succinic acid	Wheat	3.82 (3.57; 4.08)	3.82 (3.56; 4.09)	3.81 (3.55; 4.1)	0.5560	0.9472
	Rye	3.81 (3.58; 4.06)	3.91 (3.66; 4.18)	3.80 (3.54; 4.07)		
Valeric acid	Wheat	0.24 (0.21; 0.28)	0.21 (0.18; 0.24)	0.22 (0.19; 0.26)	0.2574	0.6612
	Rye	0.20 (0.18; 0.23)	0.22 (0.19; 0.26)	0.20 (0.17; 0.23)		
Isovaleric acid	Wheat	1.08 (0.98; 1.18)	0.93 (0.83; 1.04)	0.98 (0.88; 1.08)	0.7735	0.8242
	Rye	0.94 (0.86; 1.02)	0.86 (0.77; 0.96)	0.94 (0.86; 1.04)		
Caproic acid	Wheat	0.47 (0.42; 0.52)	0.45 (0.4; 0.5)	0.47 (0.42; 0.53)	0.5455	0.3119
	Rye	0.43 (0.39; 0.47)	0.45 (0.4; 0.5)	0.42 (0.37; 0.47)		

* Geometric mean and 95%, not adjusted for baseline, back transformed from log scale. ** Difference between the groups at week 6/week 12 in a baseline adjusted model.

## Data Availability

Data from this study are not publicly available as the data contain person sensitive information; data are available from the corresponding author upon reasonable request.

## Supplementary Materials

The following supporting information can be downloaded at: https://www.mdpi.com/article/10.3390/nu14081669/s1; Supplementary File S1 (Figure S1: Design of the RyeWeight study. Figure S2: Flow chart of participants in the RyeWeight study. Figure S3: Alpha diversity. Table S1: Overview of intervention products. Table S2: Composition of intervention products. Table S3: Bacteria that differed between treatments before corrections for multiple testing. Table S4: Correlations between changes in SCFA and changes in metabolic risk markers and anthropometric measures) and Supplementary File S2 (full correlation matrix of baseline abundance of bacteria and changes in anthropometric measures and metabolic risk markers).
