# Supplementary material for: The Effects of High Fiber Rye, Compared to Refined Wheat, on Gut Microbiota Composition, Plasma Short Chain Fatty Acids, and Implications for Weight Loss and Metabolic Risk Factors (the RyeWeight Study)
Source: Nutrients. 2022 Apr 17;14(8):1669. doi: 10.3390/nu14081669 (PMC9032876; doi:10.3390/nu14081669)
Supplement: Supplementary file 1 [file nutrients-14-01669-s001.zip › nutrients-1668499-supplementary.pdf]

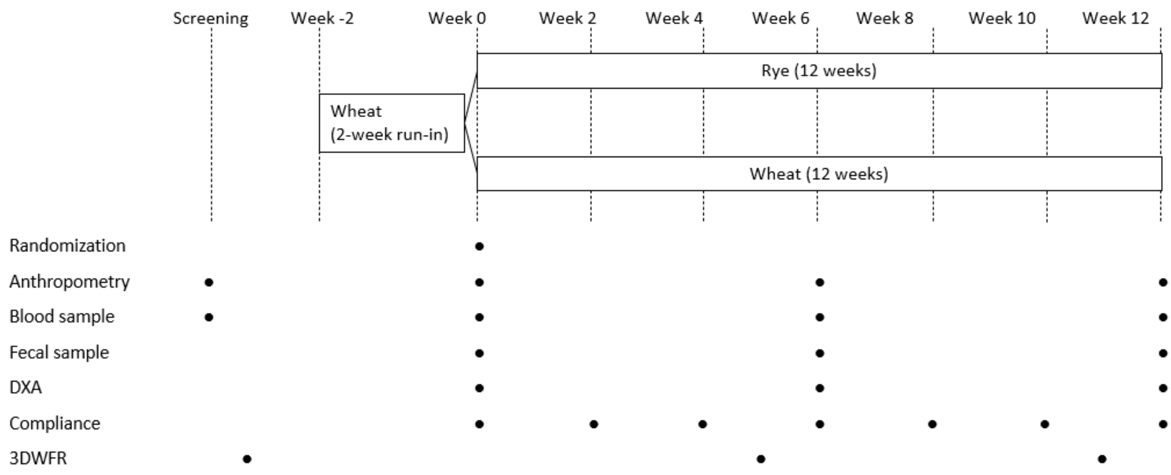

Supplemental figure S1: Design of the RyeWeight study. Abbreviations: DXA, dual-energy x-ray absorptiometry; 3DWFR, 3-day weighed food record.

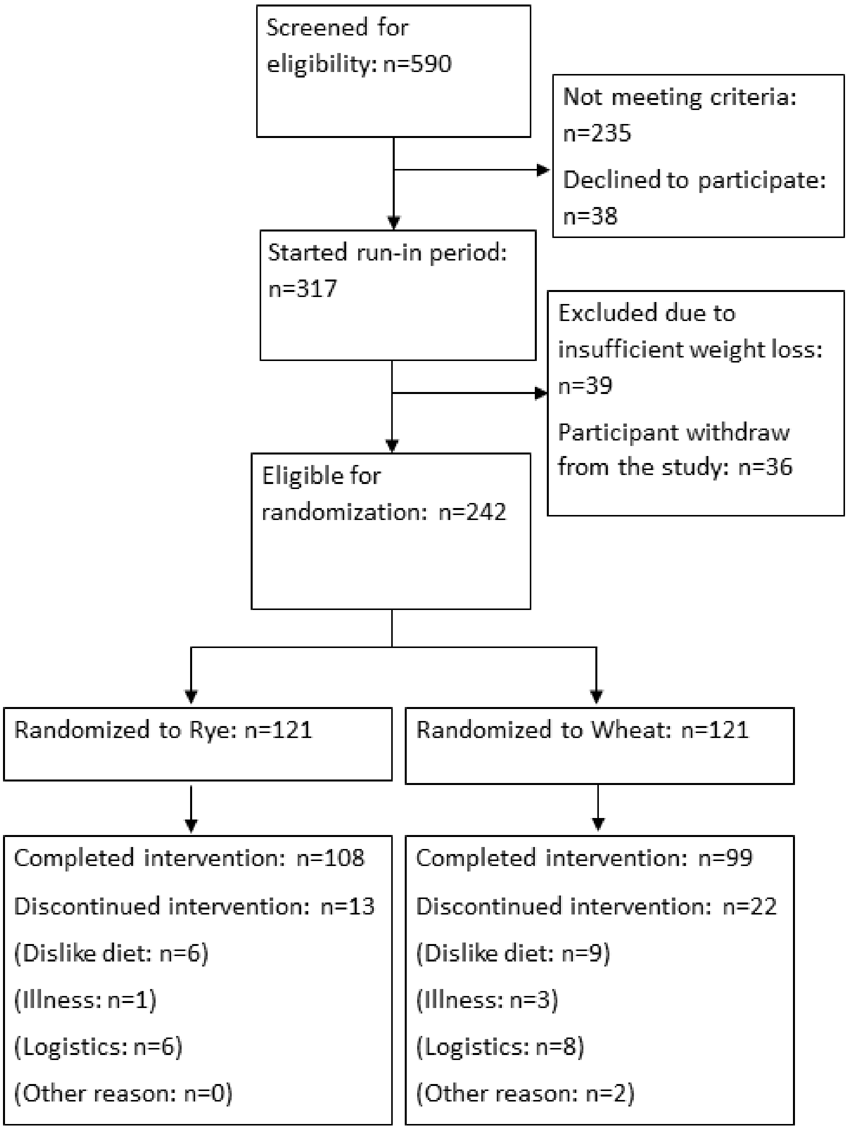

Supplemental figure S2: Flow chart of the participants in the RyeWeight study

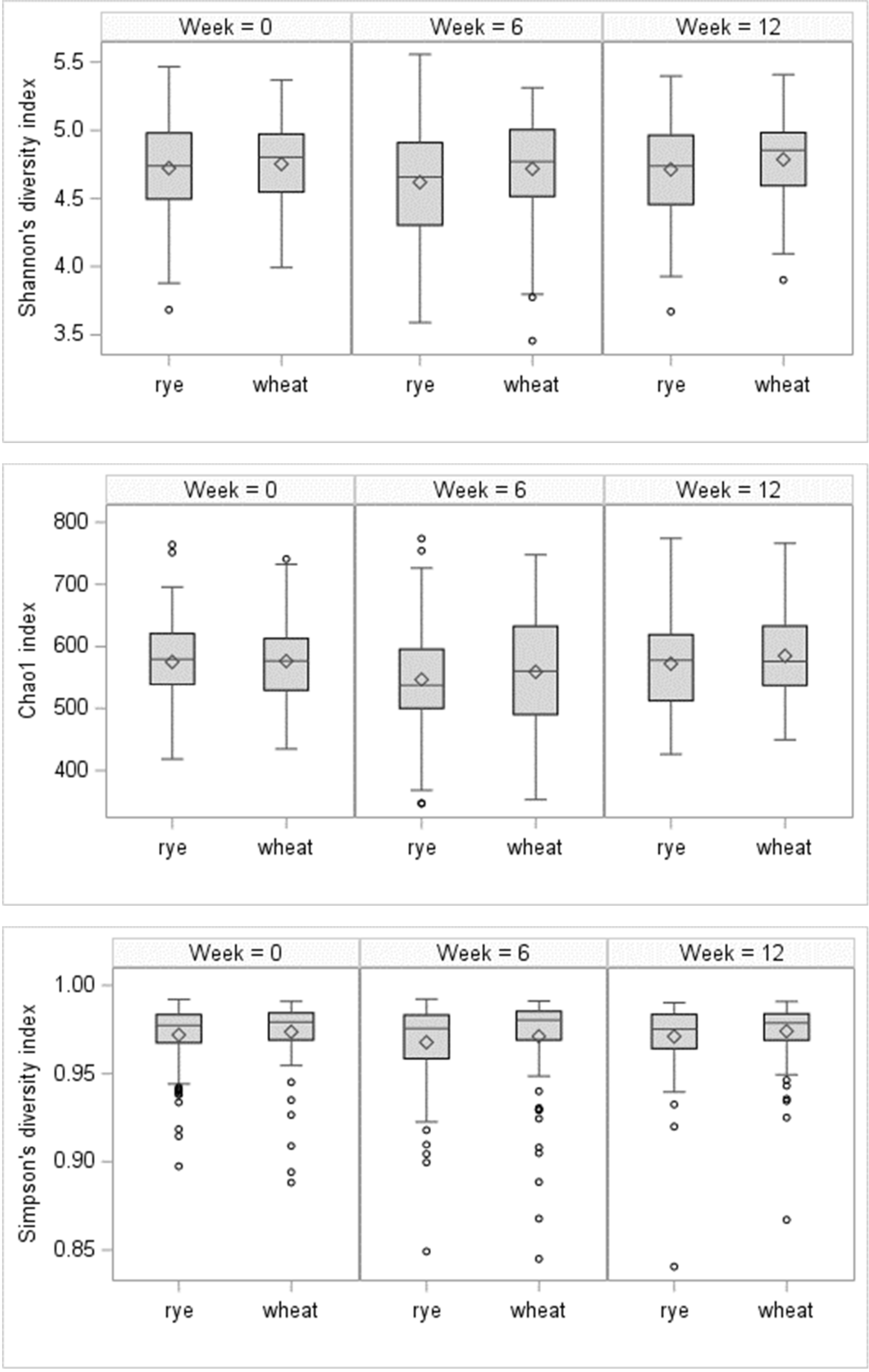

Supplemental figure S3: Alpha diversity, shown as Shannon's diversity index (top), Chao1 index (middle) and Simpson's index (bottom) in the rye and the wheat groups at week 0, week 6 and week 12. There was no significant difference between the groups at any time point (p>0.05).

Supplemental table S1: Overview of the intervention products used in the RyeWeight study

	**Cereal ingredients**	**Fermentation (breads)**	**Supplier (Commercial name)**
Extruded rye puffs	Whole grain rye flour (100% of flour weight)	-	Lantmännen (-*)
Rolled rye flakes	100% wholegrain rye	-	Lantmännen (AXA Rågflingor)
Rye crisp bread 'Rågi'	Whole grain rye flour (73% of flour weight), sifted rye flour (27% of flour weight)	Yeast	Barilla (Wasa Rågi)
Rye crisp bread 'Husman'	Whole grain rye flour (100% of flour weight)	Yeast	Barilla (Wasa Husman)
Rye crisp bread 'Sport'	Whole grain rye flour (100% of flour weight)	Yeast	Barilla (Wasa Sport)
Rye crisp bread 'Delikatess'	Whole grain rye flour (100% of flour weight)	Unfermented	Barilla (Wasa Delikatess)
Soft rye bread	Whole grain rye flour (100% of flour weight)	Yeast	Lantmännen (-*)
Extruded wheat puffs	Sifted wheat flour (100% of flour weight)	-	Lantmännen (-*)
Wheat semolina	100% Sifted wheat	-	Lantmännen (AXA mannagryn)
Wheat crisp bread	Sifted wheat flour (100% of flour weight)	Yeast	Barilla (Wasa Frukost)
Soft wheat bread	Sifted wheat flour (100% of flour weight)	Yeast	Lantmännen (Schulstad Sandwino Sub)

*Product was developed and produced for the study and is not commercially available.

Supplemental table S2: Composition of intervention products, per 100 g edible product. Participants were instructed to consume 60 g breakfast cereals (puffs or flakes/semolina), 4-6 slice of crisp bread (53-66g) and one serving of soft bread (Wheat: 70g, rye: 119g) per day, why the intake as per protocol varied depending on the participants choice of crisp bread and breakfast cereals.

	Product weight (g)	Energy (kcal)	CHO (g)	Protein (g)	Fat (g)	Dietary fiber (g)†			Arabinoxylans (g)			Fructans (g)	Klason lignin (g)	Glucose (g)	Total AR (mg)	C17:0/C21:0 ratio
						Total	*Extr.*	*Unextr.*	*Total*	*Extr.*	*Unextr.*					
**Per 100g edible product**																
Rye products																
Extruded rye puffs	100	345.6	64.0	9.0	2.2	16.78	6.86	9.92	7.16	2.29	4.87	3.85	1.28	3.59	24.9	1.15
Rolled rye flakes	100	345.8	63.7	8.6	2.2	18.33	7.41	10.92	8.34	3.29	5.05	3.02	1.34	4.69	35.8	0.98
Rye crisp bread 'Rågi'	100	339.8	65.9	8.8	1.4	14.14	5.60	8.54	6.59	2.47	4.10	2.30	0.65	3.74	39.5	0.93
Rye crisp bread 'Husman'	100	335.6	63.1	9.0	1.5	16.95	6.65	10.30	8.16	2.98	5.18	2.45	1.24	4.18	39.7	1.00
Rye crisp bread 'Sport'	100	337.5	63.6	8.4	1.6	17.50	6.11	11.39	8.07	2.44	5.64	2.61	1.09	4.80	43.3	0.97
Rye crisp bread 'Delikatess'	100	336.1	61.0	10.0	1.5	19.27	7.39	11.88	8.43	2.54	5.89	3.79	1.19	4.96	38.3	0.97
Soft rye bread	100	225.6	34.4	7.5	4.1	10.62	3.75	6.87	4.54	1.50	3.04	1.52	1.04	2.92	32.9	0.86
Wheat products																
Extruded wheat puffs	100	367.7	73.4	11.5	1.9	5.42	2.51	2.91	2.36	1.24	1.12	1.05	0.78	0.75	0.8	nd**
Wheat semolina	100	353.5	75.0	10.0	0.8	2.95	1.33	1.63	1.17	0.56	0.61	0.62	0.17	0.54	nd*	nd*
Wheat crispbread	100	392.4	65.6	12.4	7.7	5.56	1.52	4.04	2.56	0.86	1.69	0.39	0.50	1.50	4.1	0.05
Soft wheat bread	100	252.2	43.3	13.7	1.9	3.53	1.03	2.50	1.24	0.76	0.49	0.15	0.71	1.05	1.6	nd**
**Average daily amount of intervention products prescribed per protocol**																
**Rye** Mean	235	664	114.6	19.2	7.0	32.7	12.3	20.4	14.4	4.9	9.5	5.4	2.6	8.4	80.01	0.93
(minimum)	(232)	(656)	(112.5)	(18.8)	(6.9)	(30.2)	(11.5)	(18.7)	(13.2)	(4.5)	(8.7)	(4.8)	(2.3)	(7.6)	(75.00)	(0.91)
(maximum)	(239)	(679)	(117.5)	(19.8)	(7.2)	(34.2)	(12.9)	(21.6)	(15.2)	(5.4)	(10.0)	(6.2)	(2.7)	(9.2)	(86.62)	(0.95)
**Wheat** Mean	196	652	118.1	24.2	7.2	8.7	2.9	5.8	3.6	1.6	2.0	0.9	1.1	2..1	4.09	0.03
(minimum)	(196)		(117.6)				(2.5)						(0.9)		(3.86)	(0.03)

(maximum)	(196)	(648)<br>(656)	(118.6)	(23.8)<br>(24.7)	(6.9)<br>(7.6)	(7.9)<br>(9.4)	(3.2)	(5.4)<br>(6.2)	(3.3)<br>(4.0)	(1.4)<br>(1.8)	(1.8)<br>(2.1)	(0.7)<br>(1.0)	(1.3)	(2.0)<br>(2.2)	(4.33)	(0.04)

†Including arabinoxylans, fructans, klason lignin and glucose, *no alkylresorcinol detected in the product, **no C17:0 detected in the product. Abbreviations: CHO, carbohydrate; extr, extractable, unextr, unextractable; AR, alkylresorcinols.

Supplemental table S3: Bacteria that differed in abundance between the groups at week 6 and/or week 12 before FDR correction, but not after (bacteria that differed even after FDR are shown in results section of the paper).

		**Week 0***	**Week 6***	**Week 12***	p-value, raw**		p-value, FDR**	
					**Week 6**	**Week12**	**Week 6**	**Week 12**
Flavonifractor	Wheat	0.049 (0.038;0.064)	0.033 (0.024;0.045)	0.034 (0.025;0.045)	**0.0015**	**0.0038**	0.1545	0.3800
	Rye	0.048 (0.037;0.062)	0.017 (0.013;0.023)	0.02 (0.015;0.026)				
Romboutsia	Wheat	0.091 (0.076;0.109)	0.106 (0.088;0.128)	0.104 (0.09;0.12)	**0.004**	**0.0061**	0.3960	0.5978
	Rye	0.123 (0.103;0.146)	0.079 (0.066;0.094)	0.086 (0.075;0.1)				
Erysipelatoclostridium	Wheat	0.012 (0.009;0.016)	0.006 (0.005;0.008)	0.01 (0.007;0.013)	**0.0103**	**0.0024**	0.9373	0.2496
	Rye	0.011 (0.008;0.014)	0.004 (0.003;0.005)	0.005 (0.004;0.007)				
[Eubacterium] xylanophilum group	Wheat	0.28 (0.238;0.328)	0.34 (0.292;0.396)	0.282 (0.24;0.331)	**0.0012**	**0.0083**	0.1248	0.7802
	Rye	0.271 (0.232;0.316)	0.45 (0.389;0.521)	0.361 (0.31;0.421)				
Dorea	Wheat	0.848 (0.745;0.964)	0.865 (0.756;0.989)	0.841 (0.753;0.94)	**0.0047**	**0.0048**	0.4606	0.4752
	Rye	0.732 (0.647;0.828)	0.635 (0.558;0.722)	0.651 (0.585;0.724)				
UBA1819	Wheat	0.067 (0.056;0.079)	0.055 (0.047;0.065)	0.06 (0.052;0.069)	**0.011**	**0.0038**	0.9768	0.3800
	Rye	0.061 (0.052;0.072)	0.04 (0.034;0.047)	0.044 (0.038;0.05)				
Negativibacillus	Wheat	0.015 (0.011;0.021)	0.012 (0.009;0.017)	0.018 (0.013;0.025)	**0.0074**	**0.0065**	0.6808	0.6305
	Rye	0.011 (0.008;0.015)	0.006 (0.005;0.009)	0.009 (0.006;0.012)				
Blautia	Wheat	2.166 (1.879;2.496)	2.126 (1.843;2.452)	2.073 (1.821;2.359)	0.0741	**0.0015**	0.9958	0.1575
	Rye	1.941 (1.694;2.224)	1.72 (1.501;1.972)	1.54 (1.361;1.743)				
Collinsella	Wheat	0.289 (0.24;0.348)	0.303 (0.256;0.358)	0.464 (0.396;0.543)	**0.0065**	**0.0076**	0.6175	0.7296
	Rye	0.241 (0.202;0.289)	0.211 (0.179;0.247)	0.332 (0.286;0.386)				

Shuttleworthia	Wheat	0.011 (0.007;0.015)	0.008 (0.006;0.011)	0.005 (0.003;0.006)	**0.0248**	**0.0031**	0.9958	0.3193
	Rye	0.009 (0.006;0.013)	0.004 (0.003;0.006)	0.002 (0.002;0.003)				
Monoglobus	Wheat	0.321 (0.289;0.357)	0.35 (0.314;0.39)	0.307 (0.277;0.34)	**0.0027**	0.4912	0.2754	0.9854
	Rye	0.314 (0.284;0.347)	0.281 (0.253;0.311)	0.29 (0.263;0.32)				
Coprococcus	Wheat	1.308 (1.166;1.468)	1.527 (1.345;1.733)	1.322 (1.195;1.463)	**0.0028**	**0.0353**	0.2828	0.9854
	Rye	1.248 (1.118;1.393)	1.192 (1.056;1.345)	1.14 (1.035;1.256)				
Lactococcus	Wheat	0.003 (0.002;0.004)	0.002 (0.002;0.003)	0.003 (0.002;0.004)	**0.0313**	**0.0086**	0.9958	0.7998
	Rye	0.003 (0.002;0.003)	0.002 (0.001;0.002)	0.002 (0.002;0.002)				
Veillonella	Wheat	0.018 (0.013;0.024)	0.015 (0.012;0.02)	0.024 (0.019;0.031)	**0.0071**	**0.0206**	0.6674	0.9854
	Rye	0.023 (0.018;0.03)	0.028 (0.021;0.036)	0.039 (0.03;0.05)				
Eisenbergiella	Wheat	0.013 (0.009;0.019)	0.01 (0.007;0.014)	0.006 (0.005;0.009)	**0.0221**	**0.0077**	0.9958	0.7315
	Rye	0.016 (0.011;0.022)	0.006 (0.004;0.008)	0.004 (0.003;0.005)				
CAG_56	Wheat	0.145 (0.116;0.181)	0.203 (0.167;0.248)	0.144 (0.116;0.179)	**0.0035**	0.1831	0.3500	0.9854
	Rye	0.111 (0.089;0.137)	0.127 (0.105;0.153)	0.109 (0.089;0.134)				
Paludicola	Wheat	0.011 (0.008;0.015)	0.007 (0.005;0.01)	0.006 (0.005;0.009)	**0.0049**	0.5007	0.4753	0.9854
	Rye	0.01 (0.008;0.014)	0.004 (0.003;0.005)	0.005 (0.004;0.007)				
Bifidobacterium	Wheat	0.396 (0.319;0.492)	0.498 (0.406;0.612)	0.700 (0.582;0.842)	**0.0186**	**0.0152**	0.9958	0.9854
	Rye	0.331 (0.269;0.408)	0.606 (0.498;0.738)	0.844 (0.707;1.007)				
[Eubacterium] eligens group	Wheat	0.662 (0.558;0.785)	0.63 (0.532;0.747)	0.498 (0.434;0.571)	**0.0073**	0.0709	0.6789	0.9854
	Rye	0.648 (0.55;0.762)	0.473 (0.401;0.556)	0.421 (0.369;0.48)				
Adlercreutzia	Wheat	0.063 (0.051;0.079)	0.061 (0.048;0.078)	0.072 (0.061;0.086)	**0.0115**	0.1325	0.9890	0.9854
	Rye	0.054 (0.044;0.067)	0.039 (0.031;0.048)	0.059 (0.05;0.069)				
Streptococcus	Wheat	0.087 (0.072;0.106)	0.067 (0.055;0.081)	0.082 (0.069;0.099)	**0.0127**	0.2400	0.9958	0.9854
	Rye	0.087 (0.072;0.104)	0.049 (0.04;0.059)	0.071 (0.06;0.085)				
Sutterella	Wheat	0.789 (0.645;0.965)	0.779 (0.637;0.952)	0.927 (0.759;1.133)	**0.0108**	0.5359	0.9720	0.9854
	Rye	0.788 (0.65;0.955)	1.043 (0.86;1.264)	0.996 (0.822;1.206)				
Lachnospiraceae AC2044 group	Wheat	0.087 (0.065;0.116)	0.12 (0.088;0.164)	0.094 (0.07;0.127)	**0.0309**	**0.0231**	0.9958	0.9854
	Rye	0.088 (0.067;0.117)	0.188 (0.14;0.254)	0.151 (0.113;0.201)				
	Wheat	0.133 (0.113;0.157)	0.148 (0.126;0.172)	0.127 (0.11;0.146)	**0.0111**	0.1559	0.9768	0.9854

Lachnospiraceae UCG 004	Rye	0.142 (0.121;0.166)	0.194 (0.168;0.226)	0.147 (0.129;0.169)				
Colidextribacter	Wheat	0.151 (0.135;0.17)	0.142 (0.127;0.158)	0.165 (0.151;0.181)	0.1379	**0.0106**	0.9958	0.9646
	Rye	0.163 (0.146;0.182)	0.13 (0.117;0.144)	0.145 (0.133;0.158)				
Butyrivibrio	Wheat	0.070 (0.044;0.11)	0.044 (0.027;0.071)	0.024 (0.015;0.039)	**0.0114**	0.1673	0.9890	0.9854
	Rye	0.071 (0.046;0.111)	0.020 (0.013;0.032)	0.016 (0.01;0.025)				
Roseburia	Wheat	1.719 (1.529;1.931)	1.801 (1.587;2.044)	1.897 (1.687;2.132)	0.1122	**0.024**	0.9958	0.9854
	Rye	1.780 (1.592;1.99)	1.607 (1.424;1.814)	1.620 (1.448;1.813)				
Candidatus Soleaferrea	Wheat	0.008 (0.007;0.011)	0.005 (0.004;0.006)	0.006 (0.005;0.008)	0.2326	**0.0171**	0.9958	0.9854
	Rye	0.007 (0.005;0.008)	0.004 (0.003;0.005)	0.004 (0.003;0.005)				
Merdibacter	Wheat	0.008 (0.006;0.011)	0.006 (0.005;0.009)	0.008 (0.006;0.012)	**0.0147**	0.2419	0.9958	0.9854
	Rye	0.010 (0.007;0.014)	0.004 (0.003;0.006)	0.007 (0.005;0.01)				
Lachnospiraceae UCG 001	Wheat	0.209 (0.171;0.255)	0.224 (0.187;0.268)	0.167 (0.137;0.203)	**0.017**	0.2158	0.9958	0.9854
	Rye	0.185 (0.153;0.224)	0.163 (0.137;0.193)	0.135 (0.111;0.163)				
Incertae Sedis	Wheat	0.18 (0.16;0.202)	0.24 (0.209;0.276)	0.288 (0.253;0.328)	**0.0325**	0.1129	0.9958	0.9854
	Rye	0.178 (0.159;0.2)	0.195 (0.171;0.223)	0.249 (0.22;0.282)				
Howardella	Wheat	0.004 (0.003;0.005)	0.005 (0.004;0.006)	0.006 (0.005;0.008)	**0.021**	0.4056	0.9958	0.9854
	Rye	0.004 (0.003;0.006)	0.003 (0.003;0.004)	0.006 (0.004;0.007)				
Phascolarctobacterium	Wheat	0.124 (0.094;0.164)	0.118 (0.088;0.158)	0.166 (0.124;0.224)	0.1849	**0.0258**	0.9958	0.9854
	Rye	0.101 (0.077;0.132)	0.083 (0.063;0.11)	0.099 (0.074;0.132)				
Intestinimonas	Wheat	0.238 (0.213;0.265)	0.202 (0.18;0.227)	0.229 (0.205;0.256)	**0.0378**	0.1196	0.9958	0.9854
	Rye	0.22 (0.198;0.245)	0.167 (0.149;0.187)	0.199 (0.179;0.221)				
Oscillibacter	Wheat	0.211 (0.186;0.239)	0.191 (0.168;0.216)	0.2 (0.173;0.23)	**0.0299**	0.2037	0.9958	0.9854
	Rye	0.191 (0.169;0.215)	0.153 (0.136;0.173)	0.17 (0.149;0.195)				
Anaerostipes	Wheat	0.552 (0.478;0.637)	0.662 (0.573;0.765)	0.639 (0.562;0.726)	**0.0412**	0.2104	0.9958	0.9854
	Rye	0.498 (0.435;0.571)	0.764 (0.666;0.877)	0.684 (0.606;0.773)				
Lachnospira	Wheat	0.465 (0.409;0.528)	0.423 (0.362;0.494)	0.479 (0.42;0.547)	0.3593	**0.0432**	0.9958	0.9854
	Rye	0.425 (0.376;0.48)	0.37 (0.319;0.429)	0.388 (0.342;0.441)				

*Relative abundance in % as geometric mean and 95% CI (raw unadjusted data, back transformed from log scale). **Linear mixed model, adjusted for baseline abundance, data was log transformed before analysis. n(rye/wheat) =108/99.

Supplemental table S4: Correlations between changes in plasma short chain fatty acid concentration and changes in clinical outcomes. Data is Spearman's rho (p-value), significant correlations are highlighted in bold font.

	**ΔFormic acid**	**ΔAcetic acid**	**ΔPropionic acid**	**ΔButyric acid**	**ΔIsobutyric acid**	**ΔSuccinic acid**	**ΔValeric acid**	**ΔIsovaleric acid**	**ΔCaprionic acid**
**Rye group (n=108)**									
**Δweight**	0.073 (0.450)	-0.076 (0.437)	-0.061 (0.535)	-0.113 (0.252)	-0.040 (0.684)	-0.078 (0.421)	0.077 (0.432)	0.048 (0.626)	-0.095 (0.329)
**ΔFat mass**	0.010 (0.922)	-0.049 (0.612)	-0.149 (0.126)	-0.188 (0.055)	-0.044 (0.650)	-0.049 (0.615)	0.110 (0.260)	0.063 (0.519)	-0.125 (0.198)
**ΔFat%**	-0.040 (0.680)	-0.041 (0.671)	**-0.206 (0.034)**	**-0.193 (0.049)**	-0.061 (0.533)	-0.013 (0.897)	0.145 (0.136)	0.032 (0.740)	-0.095 (0.328)
**ΔCRP**	-0.168 (0.082)	0.107 (0.272)	0.024 (0.807)	-0.015 (0.877)	0.024 (0.805)	**-0.211 (0.028)**	0.034 (0.728)	0.052 (0.592)	-0.072 (0.458)
**ΔGlucose**	-0.013 (0.894)	-0.046 (0.640)	**0.239 (0.013)**	**0.245 (0.012)**	-0.071 (0.465)	-0.031 (0.748)	0.183 (0.060)	-0.128 (0.188)	-0.057 (0.556)
**ΔInsulin**	0.005 (0.959)	-0.043 (0.655)	0.142 (0.144)	0.080 (0.416)	-0.037 (0.704)	0.017 (0.860)	0.054 (0.582)	**-0.190 (0.049)**	-0.031 (0.749)
**Δtotal chol.**	-0.078 (0.423)	-0.177 (0.067)	-0.1412 (0.145)	-0.155 (0.114)	0.020 (0.835)	0.113 (0.243)	0.045 (0.642)	0.063 (0.518)	-0.012 (0.900)
**ΔLDL chol.**	0.135 (0.165)	-0.078 (0.421)	-0.133 (0.171)	-0.181 (0.065)	0.060 (0.543)	0.099 (0.307)	0.013 (0.892)	0.119 (0.221)	0.082 (0.398)
**ΔHDL chol.**	-0.105 (0.278)	-0.100 (0.302)	-0.124 (0.204)	-0.097 (0.327)	0.010 (0.915)	0.071 (0.466)	0.077 (0.428)	0.054 (0.580)	-0.019 (0.841)
**ΔTriglyceride**	0.027 (0.779)	**-0.221 (0.022)**	0.055 (0.572)	0.030 (0.763)	0.004 (0.965)	0.030 (0.755)	-0.057 (0.558)	-0.147 (0.130)	-0.051 (0.603)
**Wheat group (n=99)**									
**Δweight**	-0.037 (0.716)	-0.065 (0.523)	-0.015 (0.880)	0.130 (0.210)	**-0.202 (0.045)**	**-0.213 (0.035)**	-0.120 (0.237)	-0.080 (0.431)	-0.050 (0.624)

**ΔFat mass**	-0.056 (0.580)	-0.066 (0.518)	-0.060 (0.558)	0.107 (0.302)	-0.197 (0.051)	**-0.266 (0.008)**	-0.029 (0.775)	-0.078 (0.440)	-0.063 (0.534)
**ΔFat%**	-0.018 (0.86)	-0.069 (0.497)	-0.082 (0.422)	0.067 (0.516)	-0.148 (0.145)	**-0.239 (0.017)**	0.025 (0.807)	-0.072 (0.479)	-0.081 (0.426)
**ΔCRP**	-0.159 (0.116)	-0.112 (0.269)	-0.173 (0.088)	-0.043 (0.682)	0.049 (0.632)	0.007 (0.948)	-0.147 (0.147)	0.183 (0.070)	-0.011 (0.910)
**ΔGlucose**	0.065 (0.524)	-0.140 (0.166)	0.149 (0.143)	**0.202 (0.049)**	-0.003 (0.980)	0.0273 (0.789)	-0.079 (0.434)	**-0.209 (0.038)**	0.054 (0.596)
**ΔInsulin**	0.142 (0.160)	0.1763 (0.081)	**0.224 (0.027)**	0.173 (0.094)	-0.070 (0.490)	0.061 (0.548)	-0.064 (0.529)	**-0.291 (0.004)**	0.059 (0.560)
**Δtotal chol.**	0.145 (0.153)	0.0359 (0.724)	0.176 (0.0835)	0.132 (0.204)	-0.048 (0.639)	**-0.224 (0.026)**	0.135 (0.182)	-0.059 (0.560)	0.094 (0.355)
**ΔLDL chol.**	-0.015 (0.886)	0.0743 (0.465)	0.016 (0.874)	0.045 (0.664)	0.037 (0.719)	-0.100 (0.324)	0.017 (0.866)	0.115 (0.259)	0.057 (0.575)
**ΔHDL chol.**	-0.037 (0.716)	-0.065 (0.523)	-0.015 (0.880)	0.130 (0.210)	**-0.202 (0.045)**	**-0.213 (0.035)**	-0.120 (0.237)	-0.080 (0.431)	-0.050 (0.624)
**ΔTriglyceride**	-0.056 (0.580)	-0.066 (0.518)	-0.060 (0.558)	0.107 (0.302)	-0.197 (0.051)	**-0.266 (0.008)**	-0.029 (0.775)	-0.078 (0.440)	-0.063 (0.534)

Abbreviations: CRP, C-reactive protein; chol, cholesterol; LDL chol, low density lipoprotein cholesterol; HDL chol, high density lipoprotein cholesterol.
